# Providing full recovery with single‐dose intravenous reteplase in a patient presented to emergency department with acute ischemic stroke

**DOI:** 10.1002/ccr3.895

**Published:** 2017-03-17

**Authors:** Yunus Emre Özlüer, Mücahit Avcil

**Affiliations:** ^1^ Department of Emergency MedicineYüksekova State HospitalHakkariTurkey; ^2^ Department of Emergency MedicineAdnan Menderes University HospitalAydinTurkey

**Keywords:** Acute ischemic stroke, intravenous, reteplase, treatment

## Abstract

Administration of intravenous reteplase might be a choice of treatment of acute ischemic stroke.

## Introduction

Acute ischemic stroke (AIS) is a condition that prompts rapid diagnosis and treatment, as it is the fourth leading cause of death globally when considered apart from other cardiovascular diseases 1. Intravenous (IV) recombinant tissue‐type plasminogen activator (rt‐PA) administration is the mainstay of the early treatment of AIS in eligible patients 2.

Recombinant thrombolytic drugs approved for clinical use for various indications include alteplase, reteplase, and tenecteplase 3. Through the serine protease mechanism, they catalyze the conversion of plasminogen to plasmin. Alteplase was approved by the U.S. Food and Drug Administration (FDA) in 1996, and since then it has become a standard therapeutical agent in AIS.

Despite the advancements in endovascular interventions, intravascular thrombolysis is still the major treatment method for treating AIS. With technological developments, several rt‐PA drugs including reteplase are introduced. Reteplase is a fibrin‐specific, third‐generation thrombolytic drug and approved for clinical use in the management of acute myocardial infarction (AMI) in adults 4.

In a controlled study of 34 rabbits that had thrombolytic therapy one hour after embolization of the internal carotid artery, Yenari et al. tested reteplase versus alteplase 5. Alongside no increase in cerebral hemorrhage, it has been concluded that the advantage of single‐bolus dosing of reteplase warranted further evaluation for stroke treatment.

The first prospective human clinical trial tested the efficacy, and the safety of intraarterial reteplase in AIS was in 2002. With a relatively small group of 16 patients who were poor candidates for IV alteplase therapy, near complete or complete recanalization was achieved in 88% of patients 6.

Recent guidelines do not recommend IV reteplase, tenecteplase, and desmoteplase for treating AIS due to limited data 2. Here we report the successful use of IV reteplase for AIS.

## Case

A 44‐year‐old female patient presented with two and a half hours of difficulty with moving her left side and numbness in her face and left upper and lower extremities. She had a history of chronic hypertension and was prescribed 10 mg of amlodipine per day. Her blood pressure was 150/90 and heart rate was 84 bpm. ECG revealed a sinus rhythm. Her National Institutes of Health Stroke Scale (NIHSS) score at presentation was 4. On physical examination, she had left hemiparesis, with a muscle strength of 3 of 5, left‐sided neglect, and left facial droop.

Nonenhanced computed tomography (NECT) of the brain revealed no hemorrhage. The patient then was rapidly transferred to the ICU unit, and 10 units of IV reteplase were administered as a bolus injection in 2 min through a peripheral line. Her pathological findings started to improve from the beginning of the IV reteplase administration, and her NIHSS score was 0 when the administration finished. Her facial droop resolved, and she had full attention to her left side. Further, her muscle strength was restored in both the left upper and lower extremities.

A consecutive nonenhanced computed tomography of the brain on the 24th hour did not reveal any signs of hemorrhage and 100 mg of acetylsalicylic acid was administered as a maintenance treatment. On the following day, she was discharged with a Modified Rankin Scale of 0.

A follow‐up brain MRI obtained at another hospital did not reveal any signs of diffusion restriction and a Carotis Doppler Ultrasonography was normal, 72 h after IV reteplase administration. At the 1 month follow‐up, the patient had a normal neurological examination and a Modified Rankin Scale of 0.

## Discussion

Clinical trials with IV administration of reteplase in the setting of AIS is limited, and the results are conflicting. In a recent review 7, it was stated that no evidence is present showing benefit with reteplase for use in the setting of AIS. On the contrary, the Reopro Retevase Reperfusion of Stroke Safety Study (ROSIE) showed that in combination with Abciximab, intraarterial reteplase was revealed to have acceptable safety and efficacy in AIS patients within 3–24 h of symptom onset, with a disabling stroke, NIHSS score ≤16 points, and evidence of a perfusion deficit on PWI/MRI and MR angiography 8. In addition, in a novel rat embolic stroke model, Ma et al. showed that intravenously administered reteplase restored focal perfusion and reduced cerebral infarction at 2–4 h of ischemia, and they also stated that sole reteplase treatment might be beneficial in revascularization at the acceptable time window of 6 h 9. Moreover, reports about reteplase are mostly related to intraarterial or endovascular use 10, 11.

There is no MRI device in our urban settings. Thus, we could not obtain a diffusion‐weighted sequence of MRI. Nevertheless, parallel to evidence‐based guidelines, the absence of any signs of hemorrhage in the NECT of the brain was sufficient for us to initiate the thrombolytic therapy.

Moreover, our choice of reteplase was obligatory as it was the only thrombolytic agent we had in our hospital. Considering the pearls and pitfalls, the potential future morbidity of AIS leading to disability in young patients and possible costs was the primary motive of our initiation of therapy.

In the literature, we could not find a standardized dosage of IV administration of reteplase in AIS. For this reason, we were not sure of the dosage. Following the first bolus injection, instant recovery from lateralizing state and neglect of the patient's left side have led us to consider as a cessation sign, so we did not continue with another sequential 10 U of reteplase. Another factor in the suspension of the infusion was the concern of the longer half‐life (14–18 min) compared to alteplase (4–8 min), which may increase the risk of spontaneous intracerebral hemorrhage (SICH) 12.

One of the most devastating complications of thrombolytic therapy is reocclusion. This has been attributed to platelet activation 13. However, in the early experimental studies, it has been postulated that reteplase seems to cause greater ADP – and thrombin‐induced platelet aggregation and greater glycoprotein IIb/IIIa (GPIIb/IIIa) expression 14, but there are conflicting results in the literature. In a review, Moser et al. stated that double bolus (10 + 10 U) reteplase results in lower platelet aggregation compared to alteplase and streptokinase, in the early phase 1–2 h after thrombolysis in AMI 15. In a Thrombolysis In Myocardial Infarction (TIMI) 14 substudy, the results suggested that abciximab, combined with a reduced dose thrombolytic, could overcome the enhanced platelet activation and aggregation in the setting of thrombolysis for AMI 16. In addition, combined with abciximab, no significant differences in platelet aggregation have been found between full‐dose alteplase and reteplase groups.

We believe that the IV administration of reteplase in AIS might show benefit, yet data on this method are lacking, and there is no standardized therapy protocol.

## Authorship

YEÖ: examined, diagnosed and treated the patient, did the literature research and wrote the manuscript. MA: provided writing assistance and proofreading the article.

## Conflict of Interest

None declared.
